# Molecular targeted treatment in infants with central conducting lymphatic anomalies

**DOI:** 10.1007/s00431-025-06376-2

**Published:** 2025-08-21

**Authors:** Vera C. van den Brink, Lotte E. R. Kleimeier, Erika K. S. M. Leenders, Willemijn M. Klein, Willem P. de Boode, Joris Fuijkschot, Sabine L. A. G. Vrancken

**Affiliations:** 1 Department of Pediatrics, Amalia Children’s Hospital, Radboud University Medical Center, Nijmegen, the Netherlands; 2 Department of Human Genetics, Radboud University Medical Center, Donders Institute for Brain, Cognition and Behavior, Nijmegen, the Netherlands; 3 Department of Medical Imaging, Radboud University Medical Center, Nijmegen, the Netherlands; 4 Department of Neonatology, Amalia Children’s Hospital, Radboud University Medical Center, Nijmegen, the Netherlands

**Keywords:** Complex lymphatic anomaly, Central conducting lymphatic anomaly, Trametinib, Sirolimus, Congenital chylothorax, Hydrops fetalis

## Abstract

Central conducting lymphatic anomaly (CCLA) is a rare and potentially life-threatening vascular malformation characterized by impaired central lymphatic flow. Hydrops fetalis and congenital hydro-/chylothorax are common neonatal presentations; however, diagnosing CCLA poses challenges and requires advanced imaging. Management typically includes supportive therapies with limited effect, such as medium-chain triglyceride (MCT) diet, octreotide or propranolol, and thoracic drainage. Upcoming treatment options with mammalian target of rapamycin (mTOR) and mitogen-activated protein kinase (MEK) inhibitors have shown promising results in vascular anomalies driven by dysregulated PI3K/AKT/mTOR and RAS/RAF/MAPK signalling pathways. However, data on neonatal use remain scarce. This series describes infants (gestational age 29 + 3–40 + 4 weeks) with neonatal-onset CCLA treated with mTOR and/or MEK inhibitors (age IQR: 27–57 days), detailing clinical presentations, imaging, genetic findings, and outcomes. Genetic testing included germline and somatic variant analysis. Most patients underwent dynamic contrast-enhanced magnetic resonance lymphangiography (DCMRL) for diagnosis and to guide management. Pathogenic germline variants were identified in four patients; three had no genetic diagnosis. DCMRL revealed heterogeneous phenotypes; follow-up imaging showed improved lymphatic flow. Substantial clinical improvement occurred following mTOR and/or MEK inhibitor treatment (sirolimus and/or trametinib). In most cases, therapy was tapered within weeks; no relapses occurred (mean follow-up 10.3 months). No deaths or other severe adverse events occurred during inhibitor treatment.

*Conclusion*: This series describes infants with CCLA, treated with mTOR and/or MEK inhibitors early after birth, with rapid improvement possibly reflecting treatment response leading to functional recovery during a critical developmental phase of the lymphatic system.

**Table:** 

**What is Known:** *• Central conducting lymphatic anomalies are rare conditions associated with high morbidity and mortality, especially in neonates.* *• Molecular targeted therapies such as MEK inhibitors and mTOR inhibitors show promise in vascular anomalies driven by upregulated PI3K/AKT/mTOR and RAS/RAF/MAPK signalling pathways.* **What is New:** *• This series offers a detailed description of the early disease course, clinical variation, and management in infants with congenital chylothorax/hydrops fetalis due to CCLA, contributing to a better understanding of this rare condition in the neonatal period.* *• Early treatment with low-dose mTOR and/or MEK inhibitors seems effective in infants with CCLA, potentially reducing morbidity and mortality.*

**What is Known:**

*• Central conducting lymphatic anomalies are rare conditions associated with high morbidity and mortality, especially in neonates.*

*• Molecular targeted therapies such as MEK inhibitors and mTOR inhibitors show promise in vascular anomalies driven by upregulated PI3K/AKT/mTOR and RAS/RAF/MAPK signalling pathways.*

**What is New:**

*• This series offers a detailed description of the early disease course, clinical variation, and management in infants with congenital chylothorax/hydrops fetalis due to CCLA, contributing to a better understanding of this rare condition in the neonatal period.*

*• Early treatment with low-dose mTOR and/or MEK inhibitors seems effective in infants with CCLA, potentially reducing morbidity and mortality.*

## Introduction

Central conducting lymphatic anomaly (CCLA) is a subtype of a complex lymphatic anomaly (CLA), as classified by the International Society for the Study of Vascular Anomalies (ISSVA) [1]. It is characterized by impaired drainage through the central lymphatic system—including the thoracic duct and cisterna chyli—due to structural or functional abnormalities such as hypoplasia, vessel dilatation, atresia, or dysmotility. CCLA may be present in isolation or as part of a syndrome and manifests clinically with hydrops fetalis, chylothorax, chylous ascites, protein-losing enteropathy, and/or lymphedema [2]. These symptoms can cause profound nutritional and immunological compromise, and, especially in neonatal cases, severe respiratory and circulatory failure [3]. The clinical spectrum is broad, and diagnosis and management remain challenging [4].

**Fig. 1:**
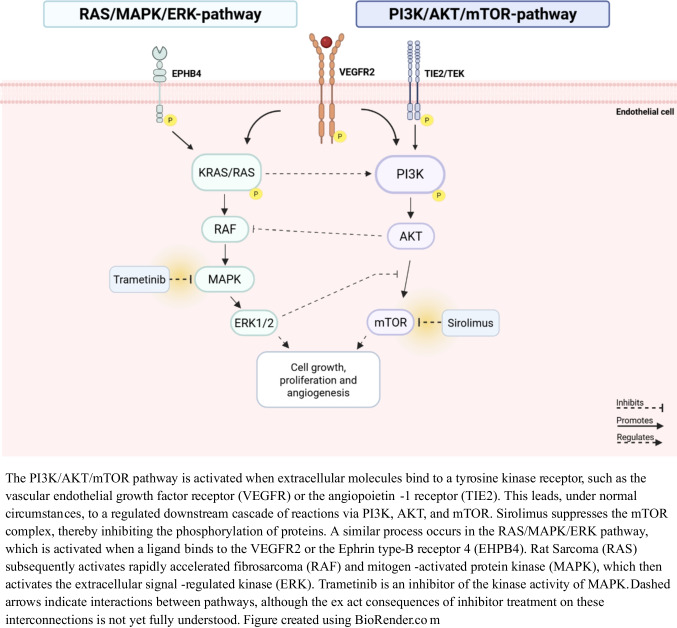
Simplified schematic overview of mTOR and RAS/MPAK pathway

Initial management is supportive, including thoracic drainage, nil-per-os (NPO), dietary modifications such as medium-chain triglyceride (MCT)-diets, and pharmacologic agents like somatostatin analogues. However, these approaches often yield limited or temporary benefit. Surgical interventions such as thoracic duct ligation have been reported in very few infants and are typically reserved for specific therapy-refractory cases [5]. Recent advances in imaging and molecular diagnostics have improved our insight into disease characteristics [6, 7]. Pathogenic variants in genes encoding key components of the PI3K/AKT/mTOR and RAS/MAPK/MEK pathways have been identified in patients with lymphatic anomalies. These pathways regulate cell proliferation, and (lymph)angiogenesis and their dysregulations have been increasingly linked to abnormal lymphatic vessel development and function [7]. Consequently, molecular targeted therapies have emerged to counteract these disruptions [8]. A schematic overview of the signalling pathways is provided in Fig. 1.

mTOR inhibitors like sirolimus have shown clinical benefit in patients with other lymphatic disorders with and without an identified pathogenic variant, improving fluid balance, symptoms, and quality of life [9, 10]. MEK inhibitors, including trametinib, are being investigated in patients with a pathogenic variant leading to RAS/MAPK hyperactivation and have yielded promising early results [11, 12]. These therapies are now considered in CCLA, especially in severe or treatment-refractory cases. This case series presents seven infants with CCLA treated with inhibitors (sirolimus and/or trametinib). We describe their clinical, genetic, imaging, and short-term follow-up findings to support future management and improve understanding of this rare neonatal condition.

## Materials and methods

This prospective case series included infants diagnosed with neonatal onset CCLA between February 2024 and February 2025. All presented with congenital chylothorax in the peri- or postnatal period. Initial management took place in referring hospitals, following national standard treatment recommendations, including MCT diet, total parental nutrition (TPN), thoracic drainage, and, in some cases, octreotide and/or propranolol. Infants with therapy-refractory chylothorax were subsequently transferred to the study centre for advanced diagnostic evaluation and additional tailored treatment, including treatment with mTOR or MEK inhibitors. The diagnosis of CCLA was based on clinical presentation, radiologic imaging using dynamic contrast-enhanced magnetic resonance lymphangiography (DCMRL), and/or pre- or postnatal genetic testing. The DCMRL was used to assess the anatomy and flow of the central lymphatic vessels, including the thoracic duct, as well as to identify effusion sites and any cystic malformations. Genetic testing included whole exome sequencing (WES) or whole genome sequencing (WGS) and targeted next-generation sequencing (NGS) to detect somatic variants associated with vascular malformations. Inhibitor treatment was initiated following a multidisciplinary risk–benefit assessment. Trametinib was started at a low dose (0.01/kg/day), given the absence of established dosing guidelines for lymphatic disease in neonates. Sirolimus was dosed based on therapeutic drug monitoring (TDM). Parents were counselled regarding treatment rationale and potential adverse effects. Alerts were placed in the medical records to ensure vigilance for adverse events, including gastrointestinal and dermatologic complications, during treatment. Clinical data were collected on patient characteristics, diagnostics, treatment course, and short-term outcomes, including pleural fluid output, time to thoracic drain removal, respiratory support, and radiological follow-up. The Medical Ethical Committee granted a waiver under the Medical Research Involving Human Subjects Act (WMO: case number 2023–16807). Informed consent was obtained from all parents or legal guardians. Patients 2 and 3 in this cohort correspond to cases 7 and 8, which were previously described in a recently published article by Leenders et al. [13].

## Results

Seven infants (female = 5) were included, with gestational ages ranging from 29 + 3 to 40 + 4 weeks. Four patients with confirmed germline pathogenic variants associated with RAS/MAPK-pathway activation received the MEK inhibitor trametinib. The remaining three, without an identified genetic cause, were initially treated with mTOR inhibitor sirolimus. Trametinib was administered as an oral suspension Spexotras®, provided through the Novartis compassionate use programme. Sirolimus was prescribed off-label and administered via a custom-made capsule dissolved prior to oral administration, as the liquid suspension contains ethanol and was therefore avoided due to its potential side effects in neonates. Patient characteristics are summarized in Table 1.

**Table 1:** Patient characteristics

	Patient 1	Patient 2	Patient 3	Patient 4	Patient 5	Patient 6	Patient 7
Prenatal intervention	None	Thoraco-amniotic shunts, corticosteroids	Thoraco-amniotic shunts	Thoraco-amniotic shunts, corticosteroids	Thoraco-amniotic shunts	None	None
Gestational age (weeks + days) at birth	37 + 2	29 + 3	37 + 1	37 + 3	36 + 2	40 + 4	38 + 3
Sex	Male	Female	Male	Female	Female	Female	Female
Birth weight (grams)	2900	1800	2750	2430	2900	3910	3250
Clinical presentation	Bilateral chylothorax	Bilateral chylothorax, skin oedema	Bilateral chylothorax	Bilateral chylothorax	Bilateral chylothorax, ascites, general oedema	Bilateral chylothorax	Unilateral chylothorax
Genetic tests prenatal	-	WES	WES	WES	WES	-	-
Genetic tests postnatal	WES + targeted NGS panel tested on buccal mucosa	-	-	Targeted NGS panel tested on buccal mucosa + re-analysis of prenatal WES data	-	WES + targeted NGS panel tested on skin fibroblasts and chylous fluid	WES
Genetic results	Gain Xp22.33/Yp11.3 *(PAR1)* (VUS)	*RIT1* c.229G > C p.(Ala77Pro) Noonan syndrome (OMIM # PS163950)	*PTPN11* c.317A > C p.(Asp106Ala) *Noonan syndrome (OMIM # PS163950)*	Not identified	*PTPN11* c.188A > G p.(Tyr63Cys) *Noonan syndrome (OMIM # PS163950)*	Not identified	*PTPN11* c.922A > G p.(Asn308Asp) *Noonan syndrome (OMIM # PS163950)*
DCMRL findings	TD full length enhancement, obstructed lymph flow at the TD outlet; retrograde pulmonary flow	Subcutaneous, mesenterial, periportal and hilar oedema, ascites; no enhancement of TD or retroperitoneal lymphatic tract	Not performed	Pleural and pericardial fluid and ascites. Subcutaneous, retroperitoneal and mesenterial oedema; TD full length enhancement; dermal backflow	Contrast until renal arteries, severe retrograde and dermal backflow*	Pleural fluids, mesenterial oedema; contrast until renal hilus*	Pleural fluids, ascites, periportal, mesenterial, retroperitoneal and subcutaneous oedema; enhancing retroperitoneal lymphatic vessels until cisterna chyli level; dermal backflow
Additional diagnoses prior to inhibitor treatment	-	IRDS, BPD Supravalvular pulmonary stenosis, mid-septal VSD, hydro-nephrosis, thrombo-cytopenia, mild cholestasis, congenital HCMV infection	PPHN	PPHN treated with VA-ECMO, *Candida albicans* (pleural and urinary tract) infection, central line thrombosis, hypercalcemia due to subcutaneous fat necrosis	PPHN, cystic and hemorrhagic brain lesions, digital necrosis from air embolisms	-	Diffuse bowel hypoperfusion, VSD, peripheral pulmonary stenosis, myeloproliferative disease related to NS
Prior treatment (days of propranolol use before inhibitor)	MCT diet, thoracic drains, TPN	MCT diet, thoracic drain, TPN, propranolol (18d), suppletion**	MCT diet, thoracic drains, TPN, propranolol (9d), suppletion**	MCT diet, thoracic drains, TPN, octreotide, propranolol (17d), suppletion**	MCT diet, thoracic drains, TPN, propranolol (9d), suppletion**	MCT diet, thoracic drain, TPN, octreotide, propranolol (9d), suppletion**	MCT diet, thoracic drain, TPN, suppletion**
Day of life at initiation of inhibitor	50	57	18	30	30	58	27
Kind of inhibitor	Sirolimus	Trametinib	Trametinib	Sirolimus	Trametinib	Sirolimus, trametinib	Trametinib
Possible side effects inhibitor treatment	-	-	-	-	Elevated liver enzymes without function loss Rash	-	Rash
Total duration of treatment with inhibitor	224 days	28 days	86 days	35 days	121 days	Sirolimus 63 days Trametinib 18 days Combination therapy 28 days Trametinib monotherapy ongoing	134 days

*WES*, whole exome sequencing; *VUS*, variance of unknown significance; *DCMRL*, dynamic contrast enhanced magnetic resonance lymphangiography; *IRDS*, infant respiratory distress syndrome; *VSD*, ventricular septum defect; *HCMV*, human cytomegalovirus; *BPD*, bronchopulmonary dysplasia; *PPHN*, persistent pulmonary hypertension of the newborn; *NS*, Noonan syndrome; *MCT*, medium-chain triglyceride; *TD*, thoracic duct; *TPN*, total parental nutrition

*First DCMRL at initiation of treatment

**Suppletion for chylous losses may have included: albumin, fresh frozen plasma, immunoglobulins, ringers lactate

**Fig. 2:**
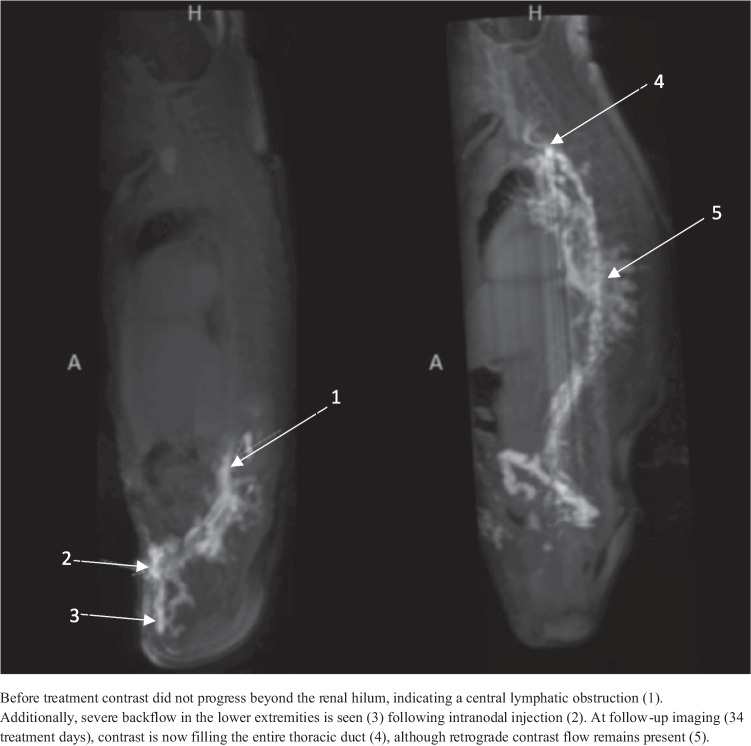
DCMRL imaging before treatment (left) (DOL27) and follow-up (right) (DOL64)

**Fig. 3:**
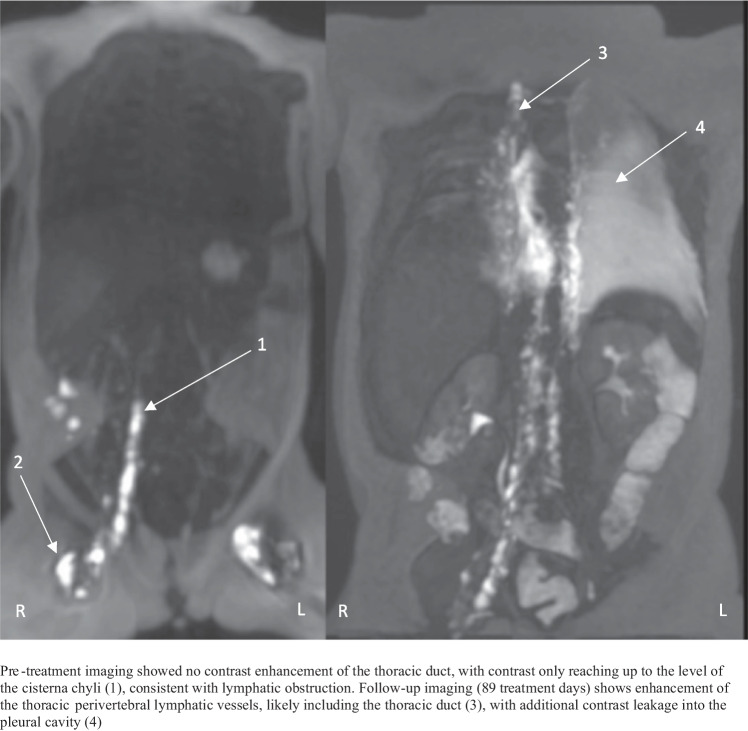
DCMRL imaging before treatment (left) (DOL56) and follow-up (right) (DOL146)

The median day of life (DOL) at initiation of inhibitor treatment was 30 (interquartile range: 27–57). Temporary treatment interruptions occurred in several patients, mostly due to central line or thoracic drain infections. Dose adjustments were made in some cases, either in response to clinical course or based on TDM (sirolimus). All patients showed clinical improvement, evidenced by decreased effusions (e.g. thoracic drain removal) and/or successful respiratory support weaning. In 6 out of 7 patients requiring thoracic drainage at treatment start, drains were removed within 5–13 days. No relapses occurred during follow-up (mean durations: 10.3 months), except for patient 6 who initially improved with drain removal after 26 days but relapsed contralaterally, requiring 42 additional days of drainage. Individual clinical courses are detailed in the case descriptions below. In two patients, follow-up DCMRL, performed to guide treatment decisions, demonstrated improved lymphatic flow with contrast now reaching the subclavian/jugular veins, compared to baseline imaging where the flow was limited to cisterna chyli level. Figures 2 and 3 illustrate these findings. No serious adverse events were observed. Reported side effects included a mild, self-limiting rash and transiently elevated liver enzymes with preserved liver function.

### Patient 1

A term male infant presented postnatally with bilateral chylothorax, for which he was treated with non-invasive respiratory support, diuretics, and a bilateral pleural puncture. Following initial stabilization, his respiratory condition deteriorated, requiring a second unilateral puncture and the initiation of NPO. DCMRL at DOL40 showed impaired drainage to the venous system, pulmonary backflow, and obstruction likely due to thoracic duct outlet valve dysfunction. SNP-array analysis revealed a copy number gain of the pseudo autosomal region 1 (PAR1) on Xp22.33/Yp11.3, including the PPP2R3B gene (i.e. arr[Hg19]Xp22.33 or Yp11.3(298440_477422) × 3). This finding is classified as a variant of uncertain significance (VUS). The clinical relevance of this variant is unknown. As surgical intervention was not feasible, sirolimus treatment was initiated on DOL50. Therapeutic blood levels were achieved by DOL63 (4–8 µg/L). Chylous effusions gradually resolved, even after the reintroduction of (MCT diet) enteral feeding, and respiratory support was discontinued at DOL82. Sirolimus treatment continued until DL224. The patient remained clinically stable, with no signs of relapse observed during follow-up at DOL411.

### Patient 2

A 29-week gestation female infant with Noonan syndrome (NS) (*RIT1*) presented antenatally with hydrops fetalis, managed with foetal bilateral thoracic shunts. Postnatally, she required invasive respiratory support due to lung hypoplasia, bronchopulmonary dysplasia (BPD), bronchomalacia, and chylothorax, treated with thoracic drainage from DOL1-14. Congenital CMV infection was confirmed retrospectively following additional testing prompted by mild cholestasis due to prolonged TPN. As the patient showed no other clinical symptoms, antiviral treatment was initially not indicated. On DOL44, DCMRL showed subcutaneous, mesenteric, periportal, and perihilar oedema and ascites with disturbed central lymphatic flow. Supportive management with corticosteroids, diuretics, propranolol, and NPO was unsuccessful. Trametinib (0.01 mg/kg/day) was started on DOL57, leading to rapid improvement and weaning from ventilation by DOL60. Trametinib was stopped on DOL83 to initiate CMV treatment, which was started later due to emerging hearing concerns. Six months later, respiratory symptoms related to BPD, lower airway malacia in combination with infections, worsened. Though there were no absolute signs of lymphatic problems (both clinically and with imaging), trametinib was restarted on DOL266 in a low dosage. With no observed clinical effect—likely reflecting the absence of lymphatic aetiology—treatment was discontinued on DOL409. The patient deteriorated due to persistent respiratory, circulatory, and neurological problems and passed away on DOL457 due to complications deemed unrelated to lymphatic disease.

### Patient 3

A term male infant was diagnosed antenatally with hydrops fetalis due to NS (*PTPN11*), for which thoracic shunts were placed. Postnatally, his condition remained critical, with persistent chylous effusions despite intensive therapy including ventilation, inotropes, bilateral thoracic drainage, octreotide, propranolol, and NPO. DCMRL was not performed due to logistical issues. Given the known activating pathogenic variance in the RAS/MAPK pathway, trametinib (0.01 mg/kg/day) was initiated on DOL18. Clinical response was favourable: thoracic drains were removed by DOL22, inotropes were stopped by DOL24, and extubation followed 2 days later. After a brief intermission due to a drain infection, trametinib was resumed at 0.01 mg/kg every 48 h. By DOL61, no respiratory support was needed. Treatment was discontinued on DOL103. At 9 months of age, the patient remains well without relapse.

### Patient 4

A term female infant was diagnosed antenatally with pleural effusions and ascites. Postnatally, she required intensive respiratory and circulatory support, and bilateral thoracic drainage. Genetic testing revealed no causative variants. On DOL6, she developed severe pulmonary hypertension of unknown origin, requiring veno-arterial extracorporeal membrane oxygenation (VA-ECMO). After decannulation, pleural effusions and generalized oedema persisted despite maximal supportive care, including ventilation, inotropes, multiple thoracic drains, one complicated by a subdiaphragmatic pleuro-visceral fistula, propranolol, and NPO. At referral (DOL28), DCMRL showed absent thoracic duct enhancement and retrograde lymphatic flow in the lower extremities. Prior to sirolimus initiation, the patient developed multiple thrombi (subclavian, jugular, cerebral venous sinuses, inferior vena cava, femoral vein), without known cause, but likely a multifactorial process. Sirolimus (0.1 mg/48 h) was started on DOL29, reaching therapeutic levels (4–8 µg/L) by DOL33. Pleural effusions resolved, with drain removal by DOL34 and fistula closure by DOL47. Weaning was prolonged due to pulmonary hypoplasia, inflammation, and muscle weakness. Sirolimus was discontinued on DOL56. She was extubated on DOL89, and respiratory support was weaned by DOL98. At 9 months of age, she remains clinically stable without relapse.

### Patient 5

A preterm female infant with a prenatal diagnosis of NS (*PTPN11*) presented postnatally with congenital chylothorax. Initial treatment included intubation and bilateral thoracic drainage. DCMRL at DOL27 showed obstruction of central lymphatic flow at the cisterna chyli. Trametinib was initiated on DOL30. Despite initial improvement, she developed extensive lymphedema, increasing respiratory needs. To guide further treatment, a repeat DCMRL on DOL64 showed marked improvement in central lymphatic flow (previously absent thoracic duct enhancement) (Fig. 2). To force further clinical improvement, trametinib was increased to 0.02 mg/kg/day, resulting in significant progress and discontinuation of respiratory support within 2 weeks. Due to elevated liver enzymes, the dose was tapered to 0.01 mg/kg/48 h, with rapid normalization of liver enzyme levels. She was discharged on DOL120, and trametinib was discontinued on DOL151. At follow-up (DOL246), she remains clinically well without signs of relapse.

### Patient 6

A term female infant presented on DOL8 with severe respiratory distress due to right-sided chylothorax, following an uncomplicated pregnancy. Initial treatment included intubation and thoracic drainage. DCMRL on DOL56 showed lymphatic flow up to cisterna chyli. Genetic testing revealed no causative variants. Sirolimus was initiated on DOL57, leading to decreased effusions and drain removal on DOL82. However, left-sided chylothorax developed shortly after, requiring drainage on DOL107. Despite high therapeutic sirolimus levels (8–10 ng/mL), the patient remained dependent on drainage, TPN, and albumin supplementation. Effusions reached up to 900 mL/day, deemed to be a mix of chyle and transudate, and drainage could not be weaned. Sirolimus was discontinued on DOL120, followed by a 14-day trametinib course (0.01 mg/kg/day), which had limited effect. A follow-up DCMRL (DOL146), performed to guide further treatment, showed improved flow up to the jugular level, and contrast leakage into the left pleural space (Fig. 3). Combination therapy with sirolimus and trametinib (0.01 mg/kg/48 h) was initiated on DOL176. After 3 weeks, attempted embolization via lipiodol injection was unsuccessful due to technical issues. The course was complicated by accidental drain removal and a *Staphylococcus aureus* empyema. Given the clinical stability, no new drain was placed, and pleural fluid accumulation remained controlled. The patient improved gradually and was discharged on DOL222 on trametinib monotherapy (0.01 mg/kg/day). At 9-month follow-up, she remained clinically stable on an alternate-day dosing schedule, without further dose adjustments for weight.

### Patient 7

A term female infant with NS (*PTPN11*) presented with a right-sided unilateral chylothorax, requiring thoracic drainage. On DOL3, pneumatosis intestinalis was observed on imaging. Laparotomy revealed diffuse bowel hypoperfusion without necrosis or perforation, and a conservative approach was taken. Cardiac, vascular, and infectious causes were ruled out. The patient experienced severe respiratory and circulatory instability, with persistent chylous effusions despite intensive therapy including mechanical ventilation, inotropes, NPO, and drainage. Trametinib (0.01 mg/kg/day) was initiated on DOL26 while on ventilation, which was weaned by DOL32. DCMRL on DOL34 showed central lymphatic flow obstruction at the cisterna chyli. The drain could be removed by DOL38. On DOL64, she underwent a laparotomy for mechanical bowel obstruction, possibly related to the earlier abdominal surgery. A second-look surgery on DOL67 required jejunal resection with anastomosis. She developed short bowel syndrome with TPN dependency and partial enteral feeding. Trametinib was adjusted to 0.01 mg/kg/48 h on DOL121. Despite ongoing enteral issues, she was thriving and discharged home on DOL132. At follow-up on DOL159, lymphatic symptoms had not recurred and trametinib was discontinued.

## Discussion

This prospective case series describes seven infants with congenital chylothorax and/or hydrops fetalis due to CCLA, all of whom were treated with either an mTOR and/or MEK inhibitor and demonstrated remarkable clinical improvement within weeks after initiation. Observations in this cohort illustrate an intriguing pattern that may reflect distinct disease courses in neonatal CCLA and warrant further investigation into timing, mechanism, and duration of targeted therapy within this population.

All infants showed clinical improvement shortly after initiation of inhibitor treatment, with tapering achievable within months and only one relapse of lymphatic symptoms to date. Notably, these improvements occurred despite low dosages and with only mild reported side effects, supporting the tolerability and potential benefit of early postnatal treatment. Compared to outcomes in older patients—where prolonged treatment, partial responses, and relapses seem more common—these observations may reflect a different therapeutic response profile in the neonatal population [14, 15].

It is hypothesized that the early postnatal period represents a phase of active lymphatic development and maturation, during which inhibitor treatment may exert enhanced modulatory effects on lymphatic vessel structure, function, and remodelling [16–18]. Consistent with this hypothesis, the observed improvements in central lymphatic flow on follow-up DCML are particularly noteworthy. Moreover, it may explain the favourable clinical trajectories in neonates treated with inhibitors. Supporting this, evidence from early studies on prenatal sirolimus use in vascular malformations, indicating that active developmental windows—even in utero—may offer therapeutic potential [19, 20]. However, comparisons between studies remain difficult due to heterogeneity in vascular anomalies, treatment protocols, and outcome measures.

Insights from developmental biology further highlight the complexity of lymphangiogenesis [21]. Although animal models have improved our understanding of lymphatic vessel formation, many regulatory pathways remain partially defined [22, 23]. Lymphangiogenesis follows distinct signalling cascades, but the full spectrum of lymphatic vessel types and their behaviour at different developmental stages is not yet fully understood [24]. Further studies are needed to clarify the molecular drivers, including mechanisms involving gene expression or epigenetic changes.

The relapse on the contralateral side following initial resolution in patient 6 remains unexplained. Prolonged drainage may have reduced afferent hydrostatic pressure and the stimulus for new vessel development, resulting in a functional impasse. There is interaction between pathways, although the impact of inhibiting one, on the activity of the other, remains uncertain. Combination therapy may have provided a more effective pathway inhibition, facilitating lymphatic remodelling. A similar approach was described by Seront et al. [25]. Additionally, residual lipiodol in collateral vessels and a pleurodesis-like response to pleural infection may also have contributed to sealing lymphatic leaks. Whether the outcome reflects the impact of treatment, impaired regeneration due to prolonged drainage, the natural disease course, or a combination thereof remains uncertain.

Three patients lacked a genetic diagnosis despite germline and (restricted) somatic variant testing, underscoring the current limitations in detecting low-level somatic mosaicism, as reported in vascular anomalies more broadly [26–28]. CCLA occurs in both syndromic and non-syndromic forms, with a growing number of variants and genes involved, reflecting its molecular heterogeneity [1, 7]. Broader gene panels and more sensitive sequencing approaches—including those using lesional tissue, lymphatic fluid, or cell-free DNA—may improve the diagnostic yield. Li et al. demonstrated that high-sensitivity sequencing across diverse tissue types can uncover variants missed by conventional methods [29]. Although favourable clinical responses were observed across patients, early genetic testing can strengthen the rationale for targeted therapy, particularly in complex refractory cases. Early identification of pathogenic variants affecting targetable pathways may enable timely, individualized treatment and enhance the understanding of disease mechanisms.

In addition to genetic testing, DCMRL proved valuable both diagnostically and, in two cases, in monitoring disease progression. Despite similar clinical presentations, lymphatic involvement patterns varied, highlighting the importance of radiological phenotyping. Follow-up DCMRL was performed in two patients and showed marked improvement in central flow, guiding treatment continuation or adjustment. However, the need for sedation and transport limits its routine use in neonates. High-resolution ultrasonography offers a promising non-invasive alternative for longitudinal monitoring [30]. Standardizing diagnostic protocols remains essential for more precise phenotyping and treatment strategies.

The small sample size and absence of a control group limit conclusions regarding the natural disease course and treatment effect. Additionally, all patients were treated with propranolol simultaneously, which may confound interpretation. Its well-established safety in children, combined with demonstrated antiproliferative and anti-lymphangiogenic effects, supports propranolol use in early management—particularly when inhibitor treatment is not yet indicated [31]. Although direct evidence is lacking, a potential mechanistic rationale supports synergism with mTOR or MEK inhibitors: by inhibiting β-adrenergic receptors, propranolol reduces VEGF expression and downstream PI3K/AKT/mTOR and RAS/RAF/MAPK pathways activation, potentially enhancing inhibitor efficacy. Further preclinical research, including in vivo models, is warranted to explore this potential interaction [24, 32]. The relatively short follow-up also limits conclusions about long-term outcomes of inhibitor treatment. The clinical heterogeneity—spanning syndromic and isolated forms—may further affect generalizability. While isolated cases of spontaneous improvement have been reported, the severity of disease in this cohort likely represents a high-risk population in whom outcomes without intervention are expected to be poor. Previous studies on congenital chylothorax suggest a mortality rate of 28% with supportive care alone [3], though comparisons are limited by genetic and phenotypic differences between patients. Nonetheless, our patients form part of an ongoing prospective cohort, enabling longitudinal analysis of disease trajectories.

This case series highlights a consistent pattern of clinical improvement in infants with CCLA following mTOR and/or MEK inhibitor treatment. These findings suggest a potentially distinct disease trajectory and therapeutic response in the neonatal population. While the mechanisms remain to be fully understood, the favourable clinical courses observed support further investigation into optimal timing, duration, and molecular targets of individualized therapy in early life. Larger studies with long-term follow-up are needed to confirm these observations and refine treatment strategies for this complex condition.

## Data Availability

The data presented in this study are available from the author upon reasonable request.

## Abbreviations

AKT: Protein kinase B; serine/threonine kinase originally identified in AKT8 retrovirus
CCLA: Central conducting lymphatic anomaly
DCMRL: Dynamic contrast-enhanced magnetic resonance lymphangiography
MAPK: Mitogen-activated protein kinase pathway
MCT: Medium-chain triglyceride
MEK: Mitogen-activated protein kinase
MEKi: Mitogen-activated protein kinase inhibitor
mTOR: Mammalian target of rapamycin
mTORi: Mammalian target of rapamycin inhibitor
NGS: Next-generation sequencing
NS: Noonan syndrome
PI3K: Phosphoinositide 3-kinase
RAF: Rapidly accelerated fibrosarcoma
RAS: Rat sarcoma
WES/WGS: Whole exome sequencing/whole genome sequencing
